# Spatiotemporal Activity Disruption of Wild Ungulates by Co‐Occurring Livestock: A Case Study in Xinjiang Kanas National Nature Reserve, China

**DOI:** 10.1002/ece3.72273

**Published:** 2025-10-07

**Authors:** Yuhang Zhu, Zhixiang Wang, Yunchuan Dai, Wancai Xia, Fan Wang, Hanlan Fei, Xueyu Wang, Dayong Li

**Affiliations:** ^1^ Key Laboratory of Southwest China Wildlife Resources Conservation (Ministry of Education) China West Normal University Nanchong Sichuan Province China; ^2^ Key Laboratory of Conservation Biology of Rhinopithecus roxellana at China West Normal University of Sichuan Province Nanchong Sichuan Province China; ^3^ Institute for Ecology and Environmental Resources Chongqing Academy of Social Sciences Chongqing China

**Keywords:** activity pattern, multi—Species occupancy model, spatiotemporal overlap, wildlife‐livestock interactions, Xinjiang Kanas National Nature Reserve

## Abstract

The spatiotemporal differentiation within habitats plays a crucial role in shaping community diversity and coexistence mechanisms. Exploring how species with similar ecological niches coexist remains a fundamental problem in ecology. We utilized infrared camera data to study the spatiotemporal activity patterns and interspecies interactions of three wild ungulates and sympatric livestock—red deer ( *Cervus elaphus* ), roe deer ( *Capreolus pygargus* ), wild boar ( *Sus scrofa* ), and cattle (*Bos taurus*)—in Xinjiang Kanas National Nature Reserve, China. By applying methods such as the multi‐species occupancy model, we explored how these species achieve coexistence through spatiotemporal niche differentiation. Results show that elevation (*β* = 0.595), aspect (*β* = 2.454), and tree density (*β* = 2.563) were key determinants of red deer occupancy; roe deer were strongly influenced by elevation (*β* = 1.789), aspect (*β* = 2.673), and slope (*β* = 0.796); wild boar occupancy increased with distance to water (*β* = 1.652) but declined with elevation (*β* = −1.567); cattle preferred lower elevations (*β* = −1.921). All three wild ungulate species positively correlate with the seasonal grazing covariate, indicating that their activities increase in summer. Their crepuscular activity patterns moderately overlap with those of cattle. The analysis of interspecies interactions reveals that cattle have a strong negative impact on red deer and roe deer (|ORsp| = 10.608 and 11.928, respectively); by contrast, the interaction between cattle and wild boar is relatively weaker. The spatiotemporal interaction analysis indicates that there is behavioral avoidance among species. Their co‐occurrence rates range from 25.9% to 51.9%, and the observed encounter intervals are longer than expected. This study emphasizes the disruptive impact of grazing and advocates taking measures such as shortening the grazing duration, limiting the grazing area, and removing physical barriers to maintain ecosystem health. Meanwhile, it is proposed to achieve long‐term conservation through interdepartmental collaboration and the establishment of monitoring systems.

## Graphical abstract

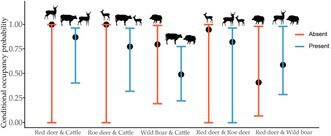

## 1 Introduction

Species coexistence, particularly among those occupying similar ecological niches, has long been a central theme in ecological research. Understanding how species achieve spatial and temporal differentiation within shared habitats is crucial for elucidating patterns of community diversity and the underlying mechanisms driving species coexistence (Chesson 2000; HilleRisLambers et al. 2012). Among sympatric species, interspecific competition occurs through both direct interference and indirect resource competition (Kronfeld‐schor and Dayan 2003), resulting in differentiation across temporal, spatial, and dietary niches. These mechanisms of niche differentiation are essential not only for explaining species coexistence but also for providing a theoretical framework for biodiversity conservation and ecosystem management (Estes et al. 2011; Ripple et al. 2014). Wild ungulates play a pivotal role in shaping ecosystem structure and functioning, serving as key bioindicators of ecosystem health (Li et al. 2022). Moreover, fluctuations in wild ungulate populations can exacerbate interspecific competition (Ripple et al. 2014; Valente et al. 2020) and influence the composition and dynamics of plant communities (Nuttle et al. 2011). Despite their ecological importance, the roles of wild ungulates in many critical protected areas remain insufficiently explored. Concurrently, rising anthropogenic pressures—including tourism and livestock grazing—are imposing additional stress on these species, posing significant challenges to conservation and management efforts (Liu et al. 2006; Zheng 2009).

Previous research on wild ungulates has predominantly focused on aspects such as anti‐predator behavior (Wikenros et al. 2015), population density estimation (Palencia et al. 2021), migration patterns (Singh et al. 2012), salt‐licking behavior (Ping et al. 2011), and habitat utilization (Bonnot et al. 2013). However, these studies have generally been limited to single‐species investigations, often overlooking interspecies interactions and large‐scale ecological dynamics. Traditional methods for observing animal behavior are frequently constrained by environmental factors, including climate and terrain, as well as inter‐individual behavioral variability, which may compromise data representativeness and lead to small sample sizes (Nathan et al. 2012). With the advancement of infrared camera monitoring technology, which offers significant advantages such as prolonged operational periods and non‐intrusive data collection, its application has expanded across various fields, including baseline wildlife surveys, behavioral ecology, population and community parameter assessments, and biodiversity monitoring (Caravaggi et al. 2017; Foster and Harmsen 2012; Li et al. 2014; Martins et al. 2007). This technology facilitates the collection of large‐scale behavioral data from multiple individuals of the same species, thereby enabling studies to transition from the individual level to the population level (Burton et al. 2015; Feng et al. 2021; Niedballa et al. 2016; Singh et al. 2022). Moreover, infrared camera monitoring has increasingly been employed to explore interspecies relationships, providing critical insights into species interactions and coexistence mechanisms within complex ecosystems (Li et al. 2024).

Xinjiang Kanas National Nature Reserve is situated in the Altai‐Sayan Mountain ecoregion (Olson and Dinerstein 2002), recognized as one of the world's 200 priority ecological areas. It is the only national‐level protected area in China located within the Euro‐Siberian biogeographic region of the Palaearctic, serving as a transitional zone between the Palaearctic and Oriental regions (Zhang 2011). This unique geographical positioning endows the reserve with distinct cold temperate coniferous forests and a continental climate. Additionally, its southern latitude combined with ample precipitation fosters high biodiversity, resulting in significantly richer taiga ecosystems compared to other regions. Consequently, the reserve represents an ideal site for investigating species spatial distribution and habitat associations (Liu 2021). Despite its ecological significance, research on the region's fauna remains sparse, highlighting the need for further scientific exploration (Cui et al. 2020). Through a comprehensive analysis of species distribution and habitat use, this study seeks to provide essential baseline data to inform and enhance conservation management efforts in this biodiversity hotspot.

Our study focused on using infrared camera data to investigate the spatiotemporal activity patterns of three wild ungulates—red deer, roe deer, and wild boar—alongside sympatric cattle in Xinjiang Kanas National Nature Reserve, China. The research aimed to evaluate how environmental factors and interspecies interactions influence species distribution and to assess the effect of cattle grazing on the spatiotemporal behavior of wild ungulates. By examining the differentiation of activity patterns under competitive and interference pressures, this study offers novel insights into ecosystem health assessment and the mechanisms underlying species coexistence. Specifically, we aimed to quantify the effects of environmental factors and grazing disturbances on the spatiotemporal activity of wild ungulates, thereby providing evidence‐based recommendations for conservation management in the region. Additionally, understanding the spatiotemporal interactions between wild ungulates and livestock will yield crucial baseline data for the prevention and control of zoonotic diseases, ultimately contributing to enhanced biodiversity conservation and improved public health management (Kukielka et al. 2013).

## 2 Materials and Methods

### 2.1 Study Area

Xinjiang Kanas National Nature Reserve shares borders with Kazakhstan, Russia, and Mongolia, and lies in the central part of the Altai Mountains in Xinjiang, China (48°3′ N–49°11′N, 86°54′E–87°54′E). The reserve has an average elevation of 1672 m, an average annual precipitation of approximately 1000 mm, and an average annual temperature of −0.2°C. During the cold season (October to March), the monthly average temperature drops to −10.4°C. The region is characterized by low thermal accumulation, a short frost‐free period (May to August), heavy snowfall, and a distinct continental climate (Liu, Gao, et al. 2020; Liu, Wang, et al. 2020). The reserve forms part of a nationally designated ecological function zone—the Altai Mountain Forest and Grassland Ecological Function Zone (Liu, Gao, et al. 2020; Liu, Wang, et al. 2020). Its vegetation predominantly consists of coniferous forests, including larch, red pine, and spruce (Zhang 2008).

In addition to its ecological importance, the reserve is a well‐known scenic tourist destination. Local herders primarily depend on tourism and livestock grazing for their livelihoods, with cattle being the dominant livestock species, followed by horses and a small number of sheep. Field surveys indicate that the study area functions as a summer grazing ground, with large herds of cattle entering the reserve in May and leaving by September.

**FIGURE 1:**
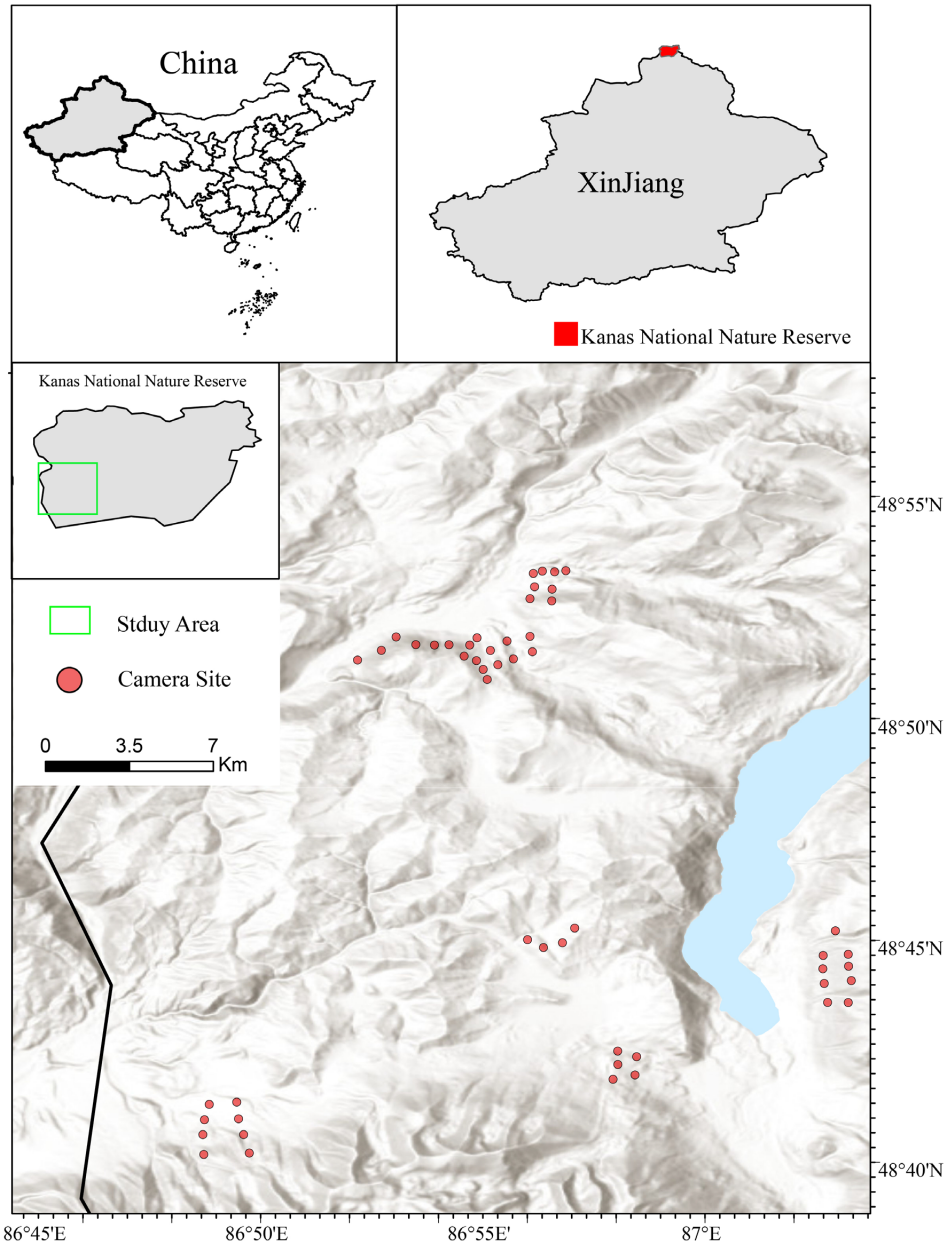
Camera trap locations in Xinjiang Kanas National Nature Reserve.

For this study, we selected a section of the southwestern experimental zone within the reserve (Figure 1). This area is notable for frequent livestock activity and occasional human presence, such as herders traversing the mountains and sporadic vehicular traffic. However, effective traffic control measures have been implemented to minimize tourist‐induced disturbances. Consequently, this zone provides an ideal setting for investigating the impact of livestock grazing as a human disturbance on the spatiotemporal activity patterns of wild ungulate species.

### 2.2 Data Collection

To focus on understanding the impact of domestic cattle on ungulates and interspecies relationships, we preferentially selected areas with livestock activity and abundant animal traces (such as animal trails, resting sites, and clear foraging marks) for placing infrared cameras. This is also a key reason for choosing the southwest region of the reserve for our study. Due to the significant landscape heterogeneity in the study area, characterized by a forest–meadow mosaic pattern, placing cameras in open meadow habitats posed challenges such as intense sunlight exposure and considerable difficulty in maintenance. Moreover, past experience indicates that this sunlight makes it difficult to capture effective photos. Therefore, cameras were primarily placed in forest habitats. Instead of using a standard grid, we adopted a random transect method along an altitudinal gradient to deploy the cameras and record environmental information at each site. Each transect is approximately 1–2 km long, and to ensure spatial independence, the distance between each camera is maintained at over 400 m. The infrared camera model we used is the LTL‐6210PLUS (manufactured by Zhuhai Lieke Electronics Co. Ltd.), configured to operate in all‐weather mode, capturing 3 consecutive photos and a 10‐s video upon each trigger. We deployed 53 infrared cameras along 15 transects and conducted regular maintenance and updates (replacing batteries and SD cards).

The collected data were processed using the Bio‐photoV2.1 plugin, resulting in a tabular dataset that recorded the camera location, date, time of day, total operational days, and other relevant information for each image set. Species identification was subsequently conducted. To ensure temporal independence of detection events, a single capture of a species by a camera was considered a valid detection. Any subsequent detections of the same species by the same camera within a 30‐min interval were regarded as a continuation of the initial event, regardless of whether the individual animal was the same (Sharma and Padmanabhan 2024; Li et al. 2016). Across the study period, the 39 cameras accumulated a total of 8120 camera‐days. Of these, 27 cameras recorded target species. Valid detection counts for wild ungulates—red deer, wild boar, and roe deer—were 548, 80, and 88, respectively. Human activity was recorded 16 times, while livestock detections included 328 for cattle, 39 for horses, and 14 for sheep. Additionally, three apex predator species were detected, including brown bears (10 detections), wolves (4 detections), and lynx (3 detections). Due to their low detection frequency, apex predators were presumed to have a limited presence in the study area and were therefore excluded from further analysis (Ciuti et al. 2012).

### 2.3 Spatial Co‐Occurrence

#### 2.3.1 Covariates

We collected a total of 10 habitat covariates, including five continuous variables: distance to the nearest road (Dtr), elevation (Ele), slope (Slo), longitude (Lon), and latitude (Lat). The categorical variables were: distance to water sources (Dtw, classified as > 100 m or ≤ 100 m), vegetation type (Vt, including mixed conifer–broadleaf forests and coniferous forests), aspect (Asp, categorized as sunny slopes, shady slopes, or flat terrain), and tree density (Td, defined as dense if canopy cover exceeded 75%, and sparse otherwise). We standardized the continuous variables (mean = 0, SD = 1) and applied contrast coding (e.g., 1, −1, 0) for the categorical variables. To avoid multicollinearity, we first calculated pairwise Pearson correlation coefficients among covariates. When absolute correlation values were greater than 0.5 (i.e., high correlation), we retained the ecologically more meaningful covariates (Tan et al. 2017). Next, we calculated the Variance Inflation Factors (VIF) for all covariates, keeping only those with a VIF below 3 (Li et al. 2022). Finally, we re‐calculated the correlations to verify the selected covariates.

#### 2.3.2 Model Construction

To analyze the spatial co‐occurrence patterns among species and the influence of environmental factors on their distribution, we employed the multi‐species occupancy model proposed by Rota et al. (Rota et al. 2016). This model extends the single‐season occupancy framework (MacKenzie et al. 2002), specifically designed to account for interactions between two or more species while addressing imperfect detection (Rota et al. 2016). The camera capture history for each survey site was divided into 10 days' detection periods, resulting in a total of 36 detection periods. If a species was detected during a given period (once or more), it was recorded as 1; otherwise, it was recorded as 0. Detection periods with missing observations were marked as NA. Additionally, since the study area serves as a summer pasture, we included the summer grazing period (time) as a detection covariate. Detection periods from May to late September, corresponding to the summer grazing season, were marked as 1, while the remaining periods were marked as 0.

We adopted a two‐step approach for model construction (Andrade‐Ponce et al. 2022). First, we developed individual species occupancy models by incorporating all covariates and calculating the Akaike Information Criterion corrected for small samples (AICc) for all candidate models (Richardson 2009). The models were then ranked based on AICc values, and model weights were calculated. Models with ΔAICc ≤ 2 were considered equivalent, and the best occupancy and detection covariates were selected from the equivalent models for each species (Guo et al. 2025). In the second step, we constructed a multi‐species occupancy model (Rota et al. 2016), specifically the interspecies interaction model 1 (M‐1), which assumes that the habitat occupancy probability of each species is influenced not only by occupancy covariates but also by interspecies interactions. The regression coefficients (*β*) in this model indicate how species respond to habitat environmental factors. A *β* > 0 signifies a positive correlation between the species' occupancy probability and the covariate, while a *β* < 0 indicates a negative correlation (Rota et al. 2016; Xiao et al. 2019). We used the “unmark” and “MuMIn” packages in R to complete the model construction and AIC calculations (Fiske and Chandler 2011; Bartoń 2013).

#### 2.3.3 The Relative Importance

To quantify the relative importance of environmental variables and interspecies interactions on each species' occupancy rate, we calculated the absolute log‐odds ratios for ORsp and ORh (Parsons et al. 2019; Rota et al. 2016). ORsp represents the odds ratio that quantifies the relationship between the presence of other species and the occupancy rate of a focal species, while holding the covariate (h) and all other environmental covariates at baseline levels (*x*). Conversely, ORh denotes the odds ratio of the predicted occupancy probability of a species in response to a change in covariate h (∆*x* units), with the absence of all other species and other covariates maintained at baseline levels.

$$\text{ORsp}=\frac{\text{odds}\left(z1=1|z2=1,z3=1,z4=1,h=x\right)}{\text{odds}\left(z1=1|z2=1,z3=1,z4=1,h=x\right)}$$

$$\mathrm{ORh}=\frac{\text{odds}\left(z1=1|z2=0,z3=0,z4=0,h=x+\Delta x\right)\;}{\text{odds}\left(z1=1|z2=0,z3=0,z4=0,h=x+\Delta x\right)}$$

In the formula, “odds” refers to the odds ratio, which is the ratio of the probability of a species occupying a given site to the probability of not occupying it (or the probability of occupying the site when covariate h changes by one unit). Here, *z* represents the species, h is the covariate, *x* denotes the value of the covariate held at baseline levels, and Δ*x* is the change in covariate h by one unit.

### 2.4 Daily Activity Patterns

We define the local sunrise and sunset times as the average time of sunrise and sunset on the first day of each month over the course of a year in the study area. We collected daily activity time records for each species based on independent detections. The recorded data were then transformed into radian‐based data, and kernel density analysis was employed to quantify the overlap of daily activities for each species. The overlap coefficient (Δ) was used to compare activity patterns between species pairs (Linkie and Ridout 2011). This coefficient quantifies the area of overlap under the density curve, with values ranging from 0 to 1. A higher overlap coefficient closer to 1 indicates a greater degree of overlap in activity patterns between two species, while a value closer to 0 suggests complete divergence. Since each species had more than 75 samples, we used the Δ4 estimator (Linkie and Ridout 2011). The overlap coefficient was categorized as follows: Δ ≤ 0.5 represents low overlap, 0.5 < Δ ≤ 0.75 indicates moderate overlap, and Δ > 0.75 signifies high overlap (Monterroso et al. 2014). The analysis was conducted in R using the “activity” and “overlap” packages (Meredith and Ridout 2016; Rowcliffe 2016).

### 2.5 Spatiotemporal Interactions

We employed the multi‐response permutation procedure (MRPP) proposed by Mielke et al. (1976) to assess the spatiotemporal separation among species (Feng et al. 2021; Karanth et al. 2017). This method examines the spatial and temporal activity patterns of the target species. We selected all camera trap sites where two or more species co‐occurred and calculated the shortest encounter time between each species pair. A dataset of these shortest encounter times was compiled for further analysis. Using 1000 simulations, we randomly assigned encounter times to the camera sites to create an expected distribution of encounter times. We then compared the actual median encounter time with this expected distribution. Under the species independence assumption, if the actual encounter time was greater than the expected distribution from the random simulations, it indicated species separation. Conversely, if the actual encounter time was shorter than expected, it suggested species aggregation. Finally, to account for potential biases in species spatiotemporal interactions caused by spatial proximity, we calculated the average of the minimum observed pairwise distances between species, as well as the mean of 1000 expected distances generated through bootstrapping.

## 3 Results

### 3.1 Spatial Co‐Occurrence

After screening, we selected seven occupancy covariates: Dtr, Ele, Slo, Dtw, Vt, Asp, and Td (Table 1). Using the single‐species occupancy model, we identified two equivalent models for each species—red deer, wild boar, roe deer, and domestic cattle—and determined their optimal occupancy and detection covariates. For red deer, the optimal occupancy covariates were elevation, aspect, and tree density. For wild boar, elevation and distance to the nearest water source were identified as the key covariates. Roe deer showed optimal occupancy based on elevation, aspect, and slope, while elevation was the sole optimal covariate for domestic cattle (Table 2). Additionally, all three wild ungulate species exhibited a positive correlation with the seasonal detection covariate of summer grazing (Table 2).

**TABLE 1:** Single species occupancy model.

Species	Model	*K*	logLik	AICc	ΔAICc	Weight
Red deer	P (Time). Ψ (Asp, Td)	5	−430.935	874.7	0	0.7
	P (Time). Ψ (Asp, Ele)	5	−431.783	876.4	1.69	0.3
Wild boar	P (Time). Ψ (Ele)	4	−234.838	479.5	0	0.435
	P (Time). Ψ (.)	3	−236.609	480.3	0.77	0.296
	P (Time). Ψ (Dtw, Ele)	5	−233.798	480.5	0.96	0.269
Roe deer	P (Time). Ψ (Asp, Ele)	5	−231.15	475.2	0	0.715
	P (Time). Ψ (Asp, Ele, Slo)	6	−230.396	477	1.84	0.285
Cattle	P (Time). Ψ (Ele)	4	−199.614	409	0	1

Abbreviations: ΔAICc, difference in AICc between the given model and the model with the lowest AICc; ψ, occupancy covariates; AICc, Akaike Information Criterion corrected for small sample sizes; Asp, aspect; Dtw, distance to water; Ele, elevation; *K*, number of parameters; LogLike, log‐likelihood; P, detection covariates; Slo, slope; Td, tree density; Time, whether each detection occasion falls within the grazing season; weight, model weight.

*** Significance is *p* < 0.001.

**TABLE 2:** Covariates influencing ungulates occupancy and detection probability according to coefficients and (*β*) associated standard errors (SE).

Species	Model component	Covariates	Estimate (*β*)	SE	*Z*	*p*
Red deer	Occupancy	Intercept	12.242	176.782	0.0693	0.9448
		Asp	2.454	1.231	1.9931	0.0463
		Ele	0.595	1.354	0.4395	0.6603
		Td	2.563	1.757	1.459	0.1446
	Detection	Intercept	−1.454	0.111	−13.09	< 0.001***
		Time	0.726	0.172	4.22	< 0.001***
Wild boar	Occupancy	Intercept	0.209	1.772	0.1179	0.9062
		Dtw	1.652	1.163	1.4203	0.1555
		Ele	−1.567	0.838	−1.8699	0.0615
	Detection	Intercept	−2.754	0.215	−12.8	< 0.001***
		Time	2.481	0.26	9.55	< 0.001***
Roe deer	Occupancy	Intercept	15.066	303.501	0.0496	0.9604
		Asp	2.673	1.233	2.1682	0.0301
		Ele	1.789	1.404	1.274	0.2027
		Slo	0.796	0.607	1.3116	0.1897
	Detection	Intercept	−2.604	0.191	−13.64	< 0.001***
		Time	1.314	0.251	5.24	< 0.001***
Cattle	Occupancy	Intercept	24.345	352.161	0.0691	0.9449
		Ele	−1.921	0.926	−2.0741	0.0381
	Detection	Intercept	−3.8	0.32	−11.88	< 0.001***
		Time	3.002	0.35	8.57	< 0.001***
Red deer: Wild boar	Occupancy	Intercept	0.296	1.136	0.2603	0.7946
Red deer: Roe deer		Intercept	−1.786	1.656	−1.0784	0.2809
Red deer: Cattle		Intercept	−10.745	176.781	−0.0608	0.9515
Wild boar: Roe deer		Intercept	−0.41	1.143	−0.3591	0.7196
Wild boar: Cattle		Intercept	−1.464	1.641	−0.8927	0.372
Roe deer: Cattle		Intercept	−12.192	303.497	−0.0402	0.968

Abbreviations: Asp, aspect; Dtw, distance to water; Ele, elevation; Slo, slope; Td, tree density.

*** Significance is *p* < 0.001.

**FIGURE 2:**
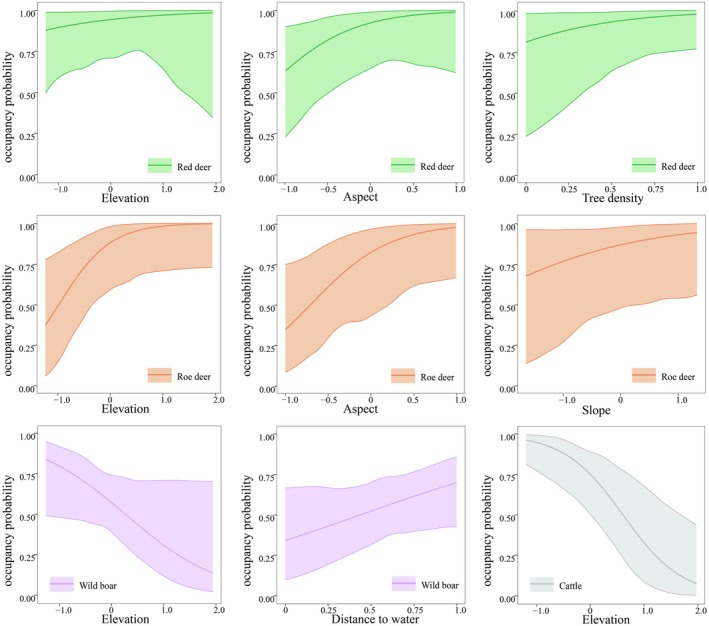
The influence of environmental variables on species occupancy rate; the shaded area represents the 95% confidence interval.

**FIGURE 3:**
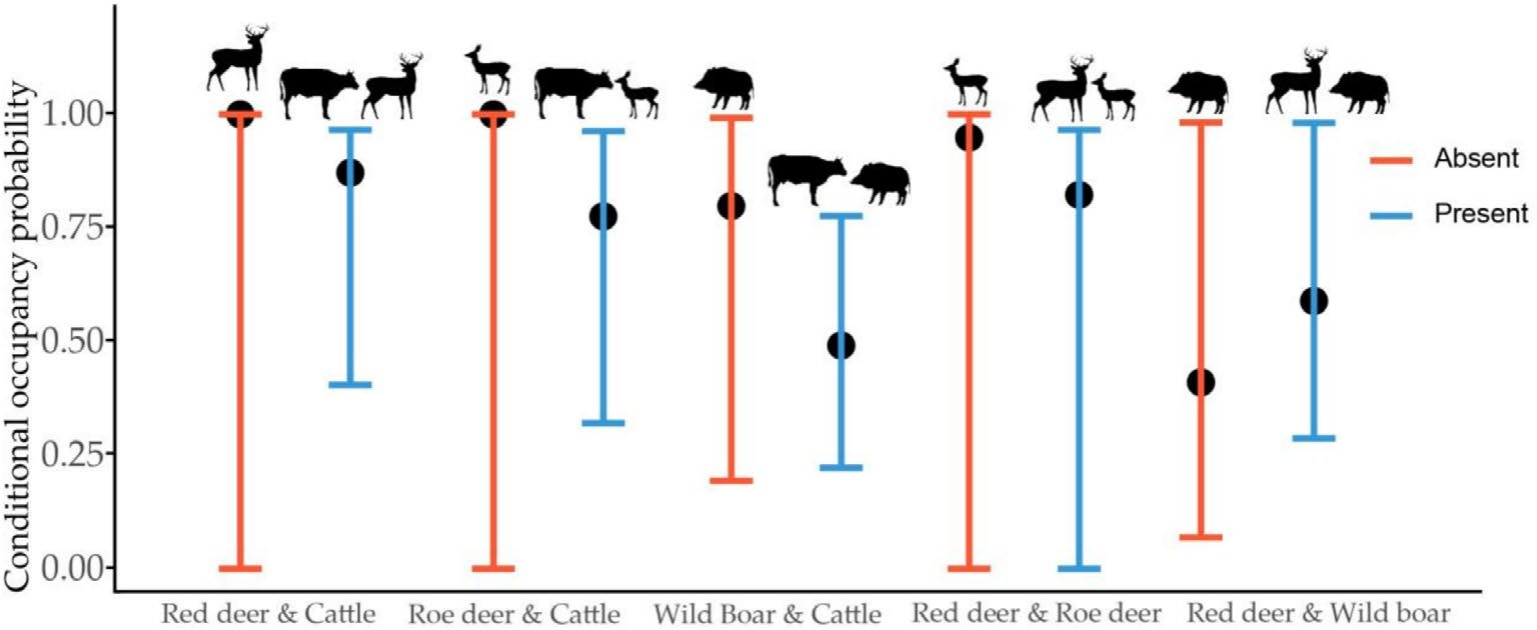
Conditional occupancy probability estimates (Pr) with 95% confidence intervals for each species in the presence and absence of dominant species (cattle and red deer).

In the multi‐species occupancy model, covariate effects varied among species: the occupancy probability of red deer increased with Ele (*β* = 0.595), Asp (south‐facing slope) (*β* = 2.454), and Td (*β* = 2.595); roe deer showed increased occupancy with Ele (*β* = 1.789), Asp (south‐facing slope) (*β* = 2.673), and Slo (*β* = 0.796); wild boar occupancy decreased with Ele (*β* = −1.567) and increased with Dtw (*β* = 1.652), favoring habitats farther from water. Cattle occupancy was negatively associated with Ele (*β* = −1.921), indicating a preference for lower elevations (Figure 2). Conditional occupancy probabilities were assessed for other species when cattle and red deer dominated: with cattle dominance, the occupancy probabilities of red deer, wild boar, and roe deer decreased (Figure 3); under red deer dominance, wild boar occupancy declined, while roe deer occupancy remained relatively stable (Figure 3).

Based on absolute log‐odds ratios (ORsp and ORh) from the multi‐species occupancy model, roe deer exhibited negative interactions with red deer and wild boar, while red deer and wild boar showed positive interactions. Cattle exhibited strong negative interactions with both red deer and roe deer, whereas the negative interaction between wild boar and cattle was weaker. Among environmental factors, Td significantly influenced red deer occupancy, Asp was key for roe deer, Dtw for wild boar, and Ele for cattle. Interspecific interactions had a stronger effect on cattle occupancy than environmental factors. Conversely, for wild ungulates, environmental factors generally had a slightly greater influence on occupancy than interspecific interactions, except for the notable interaction between wild boar and red deer (Table 3).

**TABLE 3:** The relative importance of environmental covariates and interspecific interactions in influencing the occupancy rate of the four species.

Species and environment covariates	Red deer	Wild boar	Roe deer	Cattle
*Inter‐specific interactions*				
Red deer	—	|0.7231|	|−1.3801|	|−10.6082|
Wild boar	|0.7231|	—	|−0.0266|	|−1.4104|
Roe deer	|−1.3803|	|−0.0266|	—	|−11.9276|
Cattle	|−10.6082|	|−1.4104|	|‐11.9276|	—
Elevation	|0.0680|	|−0.1098|	|0.2038|	|‐0.1809|
Aspect	|0.1950|	—	|0.2162|	—
*Influence of environmental factors*				
Distance to water	—	|0.1494|	—	—
Slope	—	—	—	—
Tree density	|0.2279|	—	—	—

### 3.2 Daily Activity Patterns

**FIGURE 4:**
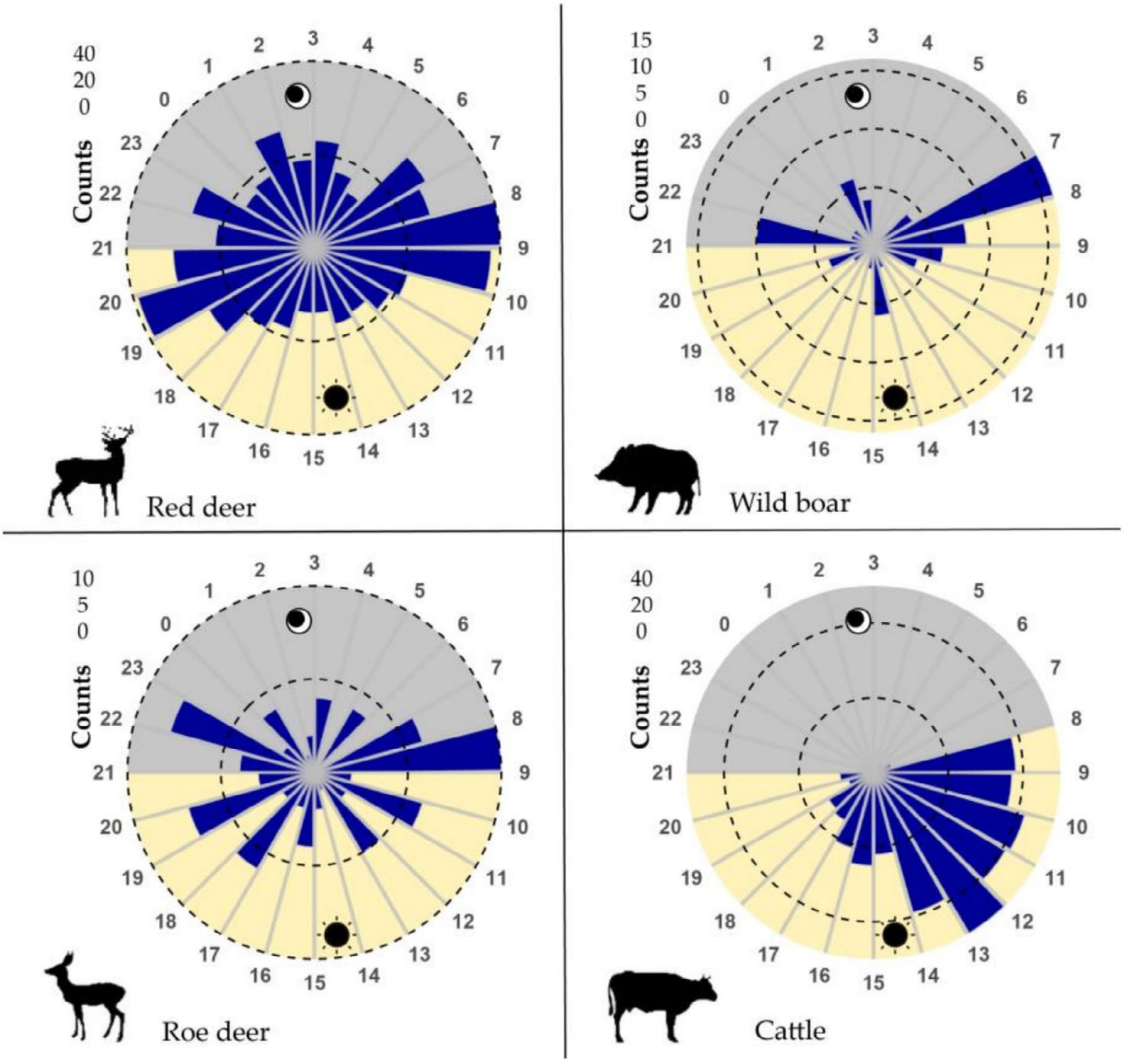
Number of records per hour for each species.

**FIGURE 5:**
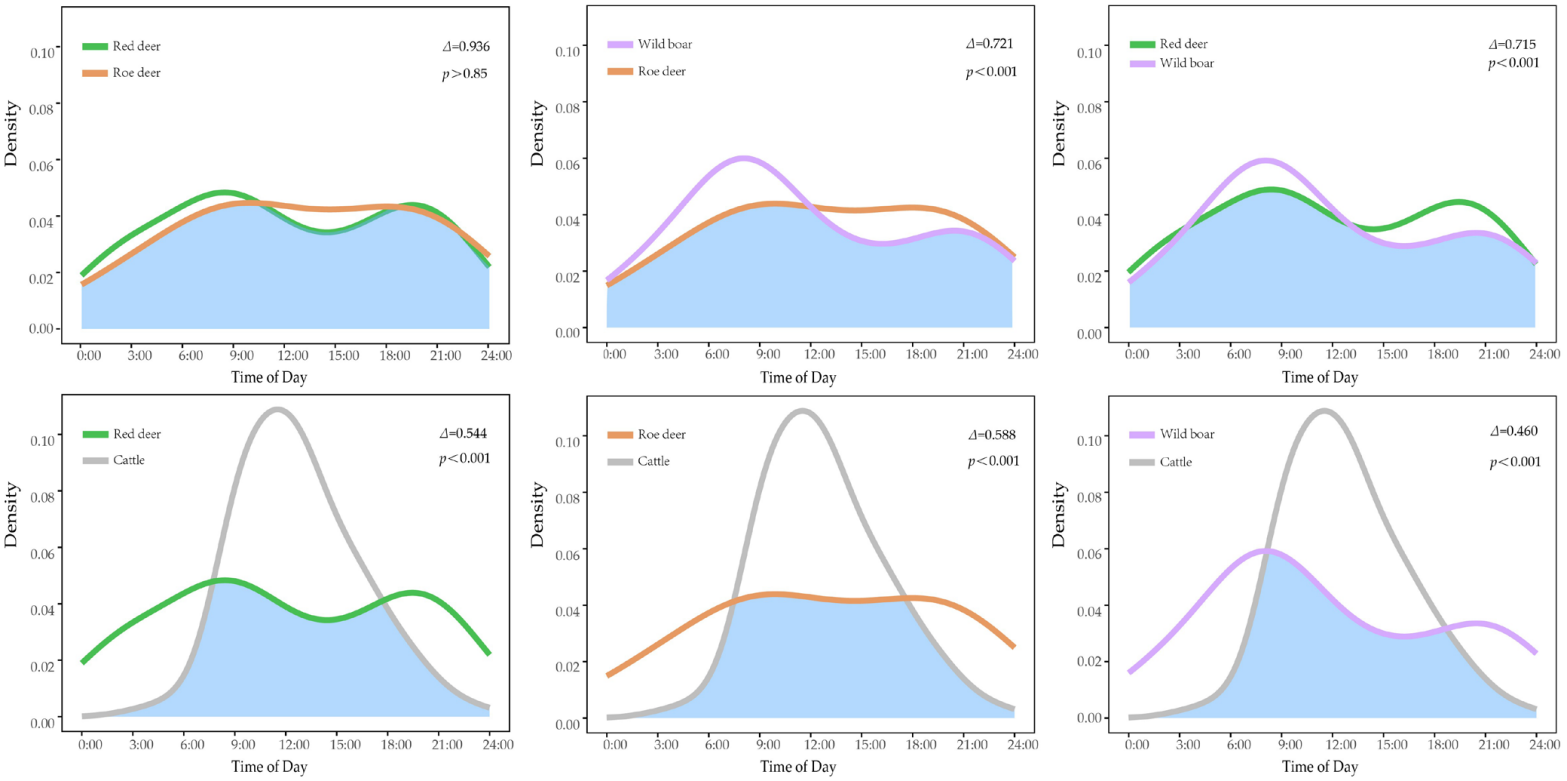
Kernel activity patterns overlap for each pair of species.

The activity patterns of red deer, wild boar, and roe deer exhibited a typical crepuscular rhythm, with activity peaks between 07:00–10:00 and 19:00–23:00 (Figure 4). In contrast, cattle displayed a diurnal activity pattern, with a distinct peak between 12:00 and 13:00 (Figure 4). Regarding daily activity overlap, cattle showed a moderate time overlap with both red deer and roe deer (0.5 < Δ ≤ 0.75; Figure 5), and the lowest time overlap with wild boar (Δ = 0.460; Figure 5). Among the three wild ungulate species, wild boar exhibited a moderate time overlap with both red deer and roe deer (0.5 < Δ ≤ 0.75; Figure 5), while red deer and roe deer displayed the highest time overlap (Δ > 0.75; Figure 5). All pairwise overlap coefficients were statistically significant (*p* < 0.001), except for the red deer–roe deer pair, which yielded a *p* value of 0.863, indicating no statistically significant difference in their activity patterns.

### 3.3 Spatiotemporal Interactions

**FIGURE 6:**
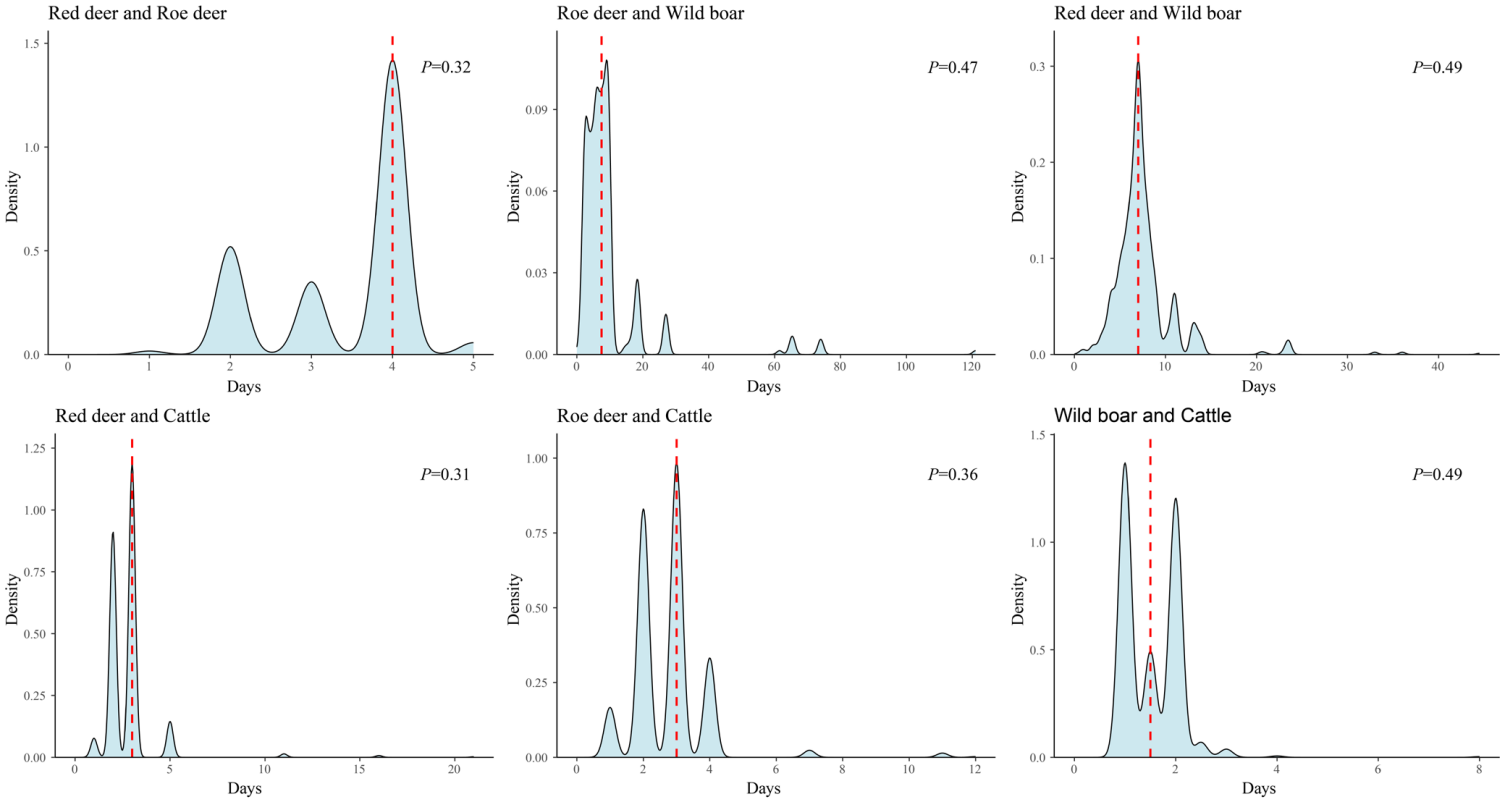
Spatiotemporal interactions among species pairs generated from multi‐response permutation procedures within the study area from June 2022 to June 2023. The density curve area represents the randomly simulated times‐to‐encounter, while the red dashed line indicates the observed median minimum time‐to‐encounter. The *p* values represent the proportion of randomly generated times‐to‐encounter values that exceed the observed times‐to‐encounter.

Following temporal and spatial overlap among species, we analyzed encounter times between species pairs to evaluate behavioral avoidance. Red deer were recorded at 24 camera stations, wild boar at 14, roe deer at 17, and cattle at 19, out of the 39 deployed cameras. Co‐occurrence at camera stations ranged from 7 to 14, representing 25.9%–51.9% of stations (Table 4). The median shortest encounter times for species pairs (1.5–7.5 days) exceeded the simulated random encounter times (1.57–6.92 days), indicating varying degrees of behavioral avoidance (Table 4, Figure 6). Subsequently, by comparing the observed mean of minimum interspecific distances with the distribution of 1000 bootstrapped expected means, we found that the majority of *p*‐values exceeded 0.05 (Table 4). This suggests that the spatial distribution of distances between wild ungulate species and livestock did not significantly deviate from random expectations, and thus, potential spatiotemporal interactions are unlikely to be confounded by spatial proximity.

**TABLE 4:** Summary of Species Encounter Time Information and Spatial Autocorrelation Validation.

Species combination	Count of camera sites with species co‐occurrences	Proportion of total camera sites with species co‐occurrence (%)	Observed median (d)	Expected median (d)	*p*	Average observed distance between species pairs	Expected average distance between species pairs	*p*
Red deer and Roe deer	14	51.90	4	3.66	0.32	629.8957	772.8322	0.124
Roe deer and Wild boar	10	37.00	7.5	6.92	0.47	1127.9241	1388.1699	0.047
Red deer and Wild boar	13	48.10	7	7.3	0.49	1694.8034	1776.2687	0.073
Red deer and Cattle	12	44.40	3	2.64	0.31	1862.9341	3313.7957	0.087
Roe deer and Cattle	7	25.90	3	2.71	0.36	2030.3371	4215.304	0.067
Wild boar and Cattle	12	44.40	1.5	1.57	0.49	807.6997	2344.0049	0.002

*Note:* Observed median refers to the Median observed minimum time‐to‐encounter (days), while expected median refers to the Expected median randomly simulated time‐to‐encounter (days). The *p* values represent the proportion of randomly generated time‐to‐encounter values that are greater than the observed time‐to‐encounter.

## 4 Discussion

This study, combining extensive animal biological data obtained from camera trap surveys with various spatiotemporal methods, provides valuable insights into the coexistence of ungulate communities in the taiga forests in Xinjiang Kanas National Nature Reserve. Despite the limited deployment of camera traps, it offers firsthand information on the spatiotemporal activity patterns of ungulate species and cattle in the region. Notably, rather than including grazing as a typical human disturbance covariate, we deliberately modeled livestock as a distinct interacting species to capture its direct influence on habitat selection and behavioral avoidance patterns.

Elevation emerged as the most influential spatial component for ungulate species in the region, with significant differences in how each species selects elevation. Wild boar and cattle prefer lower elevations, while red deer show a preference for higher elevations, albeit not strongly rejecting low elevations. Roe deer, on the other hand, significantly prefer higher elevations. Although we did not use species distribution models to directly map species' elevation zones, we hypothesize that ungulate species in this study achieve spatial niche differentiation via elevation zonation. Additionally, red deer and roe deer show a clear preference for sunny slopes, which is consistent with findings from other studies (Zhou and Zhang 2011). Roe deer, in particular, exhibit a stronger preference for sunny slopes than red deer, suggesting that besides the richer food resources on these slopes, temperature preferences may also play a role. Slope aspect can directly or indirectly reflect the influence of temperature on habitat selection (Wu et al. 2000). Our study found that roe deer preferred high‐slope habitats, which contrasts with the findings of Mori et al. (2021), who emphasized predation and environmental factors. In contrast, our analysis focuses on interspecies relationships and environmental factors. This discrepancy indicates that for roe deer, slope preferences are more related to ecological needs and habitat availability (Singh et al. 2019).

The presence of cattle influences the distribution of other species. The preference of cattle for low‐elevation habitats forces roe deer to migrate to higher elevations, consistent with the findings of Feng et al. (2021) and Stewart et al. (2002). However, contrary to these studies, wild boar in our study showed a preference for low‐elevation habitats, similar to cattle. This could be attributed to wild boar's flexible behavior, which varies according to landscape features and local conditions (Augustsson et al. 2024). The forest landscape in the study area is patchy, with higher plant species diversity at lower elevations, which may influence wild boar's preference for these areas, particularly near forest edges, aligning with their typical habitat use (Liu et al. 2019). Red deer, in contrast, have specific habitat preferences regarding canopy density, as denser habitats provide better concealment, which aligns with Kamler et al.'s (2007) observations on red deer habitat selection. Notably, wild boar in this study preferred habitats farther from water sources. This is a novel finding, and we speculate that it may be related to surrounding terrain or vegetation factors, rather than the direct distance from water. Wild boar appear to avoid steep terrain and topography.

In terms of interspecies relationships, cattle show a significant negative correlation with all three wild ungulate species, particularly with red deer and roe deer, leading to spatial coexistence via elevational segregation to avoid cattle grazing. Interestingly, the negative correlation between wild boar and cattle is weaker, with both species preferring lower‐elevation habitats. This suggests that spatial niche differentiation is not the key to their coexistence but may occur through other mechanisms, such as temporal or dietary differences. The multi‐species occupancy model indicates a slight positive correlation in occupancy between red deer and wild boar, while a slight negative correlation exists between red deer and roe deer. The conditional occupancy model further explored interspecies relationships, indicating a stronger negative correlation between red deer and roe deer, while the relationship with wild boar is more complex. Notably, the relationship between red deer and wild boar differed between the multi‐species model and the conditional occupancy model. This suggests that species interactions may change depending on the spatial scale of observation (Kneitel and Chase 2004). We favor the results from the multi‐species model, which suggest that species may select similar habitat types but avoid excessive competition through other ecological niche differentiations, consistent with the views of Feng et al. (2021).

Our study highlights that the impact of cattle grazing on wild ungulates far outweighs the effects of environmental factors and interspecies interactions. All three wild ungulate species showed a positive correlation with the detection covariate (summer grazing time), indicating a preference for activity during the summer, with reduced activity in the winter. This pattern aligns with findings by Massé and Côté (2013), suggesting that northern ungulates reduce activity in winter as an energy‐saving strategy (Moen 1976), relying on body reserves accumulated before the food‐scarce season (Cook et al. 2004; Taillon et al. 2006). This also supports the forage abundance hypothesis (MacArthur and Pianka 1966), which predicts that ungulates increase their foraging effort when forage abundance is high, thereby affecting their selectivity and movement patterns (Sæther and Andersen 1990).

The hourly activity frequency plots for each species reveal that the activity peaks of the three wild ungulate species generally coincide with sunrise and sunset. Previous studies have indicated that species' activity patterns are often influenced by sunrise and sunset times, suggesting the presence of a direct photoperiod effect (Bowland and Perrin 1995). Photoperiod is recognized as a major environmental factor affecting mammalian behavior, playing a significant role in determining activity patterns (Alves and Andriolo 2005). However, other biophysical parameters, such as temperature and precipitation, may also contribute to variations in animals' circadian rhythms to some extent (Caravaggi et al. 2018).

Our study primarily relied on camera trap data and did not account for variations due to factors such as temperature and precipitation. Nevertheless, it can be inferred that food scarcity and temperature drops could influence the timing of animal activities (Houngbegnon et al. 2020). It is generally believed that temporal adjustments allow species to minimize competition among sympatric wild ungulate species (Jia et al. 2020). However, even species with high temporal overlap often exhibit differences in their dietary patterns (Noor et al. 2017). This aligns with our findings, where there is a noticeable degree of temporal overlap among the four species, especially within the three wild ungulates. The high overlap suggests that these species may have differentiated their spatial (altitudinal) or dietary niches to facilitate coexistence. For example, red deer primarily forage on grasses, shrubs, and woody plant branches (Ligi and Randveer 2012), while roe deer feed on horsetails, pines, and grasses, including sedges and other grasses (Argunov and Stepanova 2011). In contrast, wild boars, as omnivores, have a broader range of food types (Sondej and Kwiatkowska‐Falinska 2017). The diversity of plant species reduces dietary competition, allowing these species to achieve dietary niche differentiation and coexistence. In terms of spatial coexistence, red deer and roe deer may have avoided cattle grazing disturbance through spatial niche differentiation. Interestingly, while both wild boars and cattle prefer low‐altitude areas, their activity overlap is minimal. This supports the viewpoint of Madhusudan (2004), who suggests that wild boars, as non‐ruminant omnivores, do not show a strong response to livestock activity.

Species that co‐occur in the same geographic area inevitably experience interspecific competition, which can be categorized into two types: interference competition (direct confrontation) and resource competition (consumption of shared resources) (Kronfeld‐schor and Dayan 2003). To balance the pressures of competition and the need to select suitable habitats, species often differentiate along temporal, spatial, and dietary niches (Chu et al. 2017). Our results show that the three wild ungulate species respond to cattle grazing by reducing habitat use and exhibiting behavioral avoidance. Among the wild ungulates, interspecific interactions and habitat characteristics both play crucial roles in habitat selection, as demonstrated by our multi‐species occupancy analysis. Our findings support the negative interaction between cattle and wild ungulates, suggesting that livestock may continually encroach upon the habitats of ungulate species, particularly herbivorous ruminants, or alter their ecological niches in overlapping areas (Dave and Jhala 2011; Hibert et al. 2010; Madhusudan 2004). This study did not focus on the dietary and foraging behaviors of the ungulates, and further investigation is needed to explore potential changes in their dietary niches in greater detail. Admittedly, there are some limitations to this study. Certain areas of the reserve are difficult to access due to border controls, complex terrain, and logistical constraints. As a result, fieldwork and data collection in these areas were restricted, leading to the exclusion of several important environmental covariates from the occupancy model. These missing covariates could have provided significant insights, thus making the prediction of species' habitat selection less comprehensive.

## 5 Management Implications and Recommendations

Restricting grazing could be one of the most effective strategies for protecting wild ungulate communities. The reserve supports a rich diversity of wildlife and plant species, and protecting these ecosystems not only reduces disturbance to ungulate species but also mitigates negative impacts on the entire forest habitat. This is a long‐term process that requires careful management. It is recommended that management authorities limit the time livestock are allowed in the reserve, ideally allowing grazing only during early summer when vegetation is most abundant. Alternatively, authorities could begin planning for a gradual reduction in grazing areas. In the most extreme scenario, grazing could be fully prohibited, with pastoralists guided to shift their economic activities to sustainable alternatives. Additionally, it is advised that management authorities remove the barbed wire along the reserve roads. While placed by pastoralists, these barriers severely hinder wildlife movement and interaction. Implementing these measures will require cooperation across various departments. To promote sustainability, it is crucial to establish and enforce effective monitoring, assessment, and local regulations to support the conservation of wild ungulates and their habitats.

## Author Contributions

**Yuhang Zhu:** conceptualization (equal), data curation (equal), formal analysis (equal), investigation (equal), methodology (equal), resources (equal), software (equal), validation (equal), writing – original draft (equal). **Zhixiang Wang:** data curation (equal), investigation (equal), resources (equal). **Yunchuan Dai:** software (equal), writing – review and editing (equal). **Wancai Xia:** writing – review and editing (equal). **Fan Wang:** project administration (equal), visualization (equal). **Xueyu Wang:** methodology (equal), software (equal). **Hanlan Fei:** methodology (equal), software (equal). **Dayong Li:** conceptualization (equal), funding acquisition (lead), methodology (equal), project administration (lead), resources (equal), supervision (lead), writing – review and editing (equal).

## Conflicts of Interest

The authors declare no conflicts of interest.

## Data Availability

The species occurrence data are owned by China West Normal University. Access to these data requires contacting the authors or the corresponding author. Other data are available within the main text.
