# Correction to “Spatiotemporal Activity Disruption of Wild Ungulates by Co‐Occurring Livestock: A Case Study in Xinjiang Kanas National Nature Reserve, China”

**DOI:** 10.1002/ece3.73716

**Published:** 2026-05-29

Zhu, Y., Z. Wang, Y. Dai, et al. 2025. “Spatiotemporal Activity Disruption of Wild Ungulates by Co‐Occurring Livestock: A Case Study in Xinjiang Kanas National Nature Reserve, China.” Ecology and Evolution 15, no. 10: e72273. https://doi.org/10.1002/ece3.72273.

In subsection 2.3.3, the formulas for both ORsp and ORh contained errors in their denominators. Specifically, ORsp incorrectly used the same condition for both the numerator and denominator, and ORh similarly used the same value of h in both the numerator and denominator. The corrected formulas replace the denominators with the proper reference conditions: for ORsp, the denominator now uses *z*_2_ = 0, *z*_3_ = 0, *z*_4_ = 0, *h* = *x*; for ORh, the denominator now uses *h* = *x* while keeping other variables fixed. These corrections ensure that the odds ratios accurately reflect the intended comparisons. It should be noted that this error was limited to the formula expressions in the manuscript due to our own oversight. Since the calculations were performed using R, the computational results were unaffected and remain correct.

The correct formulas are as follows:

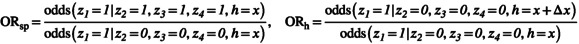




We apologize for this error.

